# Metagenomic, organoleptic profiling, and nutritional properties of fermented kombucha tea substituted with recycled substrates

**DOI:** 10.3389/fmicb.2024.1367697

**Published:** 2024-05-30

**Authors:** Suriyapriya Selvaraj, Kalaichelvan Gurumurthy

**Affiliations:** ^1^School of Biosciences and Technology, Vellore Institute of Technology, Vellore, Tamil Nadu, India; ^2^VIT School of Agricultural Innovations and Advanced Learning, Vellore Institute of Technology, Vellore, Tamil Nadu, India

**Keywords:** biomass, fermentation, kombucha tea, metagenomics, metabolites, nutritional value, sensory evaluation

## Abstract

Kombucha fermentation yields a diverse range of beneficial macro and micronutrients. In our study, we examined the metabolites, antioxidant activity, organoleptic characteristics, and nutritional attributes of traditionally prepared kombucha tea, using black tea and sugar (control) as substrates, and compared them with tea made from tea dust and blackstrap molasses (test). Kombucha tea crafted from functional raw materials exhibited enhanced sensory qualities and improved health-promoting properties. The levels of tannins, flavonoids, and phenols play a crucial role in determining the antioxidant activity of kombucha tea. Using the DPPH and FRAP methods, we investigated the antioxidant activity throughout the fermentation period, ranging from day 0 to day 12, under optimized conditions. The results consistently demonstrated an initial increase in antioxidant activity from day 0 to 6, followed by a decline from day 6 to 12. Notably, statistical analysis revealed that the antioxidant activity of the test sample was significantly better (*p* > 0.001) compared to the control sample. The nutritional content of the kombucha from day 6 of the test sample is higher than the control sample provided sugars (fructose 0.4 ± 0.1, glucose 0.7 ± 0.1, sucrose 1.4 ± 0.1) g/100 mL, minerals (calcium, 19.4 ± 0.15, iron 23.1 ± 0.25, and potassium 28.3 ± 0.25) mg/100 mL, vitamins (B1 0.58 ± 0.01, B2 0.30 ± 0.02, B3 0.33 ± 0.02, B6 0.75 ± 0.02, B9 0.19 ± 0.03, B12 0.9 ± 0.03, and C 1.38 ± 0.06) mg/100 mL, sodium 4.35 ± 0.25 mg/100 mL, calories 14.85 ± 0.25 mg/100 mL, carbohydrates 3.135 ± 0.12, and acids (acetic acid 4.20 ± 0.02, glucuronic acid 1.78 ± 0.02) mg/100 mL on day 12. The predominant microbial species identified in both control and test samples included *Komagataeibacter rhaeticus, Gluconobacter oxydans, Brettanomyces bruxellensis*, and *Zygosaccharomyces bailli*, each with varying dominance levels. These microorganisms play essential roles in metabolizing sugars, generating acids, and contributing to the distinctive flavor profile of kombucha. Sensory evaluations of the control and test samples were analyzed, and the overall preference was 88% for the test sample with tea dust and molasses. The sensory characteristics of the test sample included a fruity smell (41%), fizzy texture (66%), bright color (47%), and a fruity taste (67%), with overall acceptability (56%) rating it as excellent. Our research contributes to a deeper understanding of the interplay between raw materials, microbial composition, and the resulting composition of bioactive compounds.

## 1 Introduction

Kombucha is a fermented tea that is made by fusing sweetened black tea with a cellulose mat called SCOBY, which is a symbiotic consortium of various strains of bacteria and yeast. It is consumed for its health benefits and therapeutic properties. Kombucha is traditionally prepared by steeping black tea leaves in hot water. Sugar is added as a sweetener and for the growth of the microbes in the culture. A symbiotic consortium of bacteria and yeast (SCOBY) is the starter culture, which is inoculated into the sweetened tea and allowed to ferment. The kombucha tea is best brewed in a glass container that is covered with a muslin cloth and secured with rubber bands. It is left to ferment for 7–10 days at room temperature. Fermented tea has an impact on the intestinal microbiota and is rich in bioactive compounds and antioxidants (Jayabalan et al., 2014). The Department of Consumer Affairs price monitoring unit has listed the price for sugar as 0.54 USD/kg and for black tea powder as 3.37 USD/kg. The Ministry of Petroleum and Natural Gas has set the price for blackstrap molasses at 0.36 USD/L. Blackstrap molasses and tea waste are considered by-products of the agro-industrial industry and are used as carbon and nitrogen sources, respectively. These byproducts are produced in larger quantities and can be utilized as the growth medium for kombucha production, and they are eco-friendly and cost-effective (Valli et al., 2012; Gargey et al., 2019). Blackstrap molasses is a beneficial raw material in the fermentation process as it is cost-effective and also rich in minerals, organic compounds, and vitamins (Rodrigues et al., 2006; Malbaša et al., 2008). Research by Iqbal found molasses to have significantly higher phenolic content (3,751 μg GAE/g) compared to raw sugar (27.75 μg GAE/g) and refined sugar (23.81 μg GAE/g), making them a beneficial alternative raw material for kombucha production (Iqbal et al., 2017). The quality and quantity of the starter culture, type of tea, and sugar determine the concentration and flavor of the metabolites present in the fermented tea (Jayabalan et al., 2014). Kombucha has high beneficial values and is taken as a refreshment beverage (Sreeramulu et al., 2000). Green tea, oolong tea, lemon balm tea (Četojević-Simin et al., 2012), jasmine tea, and mulberry tea (Talawat et al., 2006) were used as nitrogen sources in kombucha fermentation. By-product waste like banana peel extract (Ebrahimi Pure and Pure, 2016), soybean whey (Tu et al., 2019), and acerola (Leonarski et al., 2021) were used as raw materials for kombucha fermentation. The bacteria and yeast in kombucha utilize substrates and produce various beneficial metabolites. The metabolites of the fermented tea beverage consist of macronutrients and microelements, which were examined under static conditions (Jayabalan et al., 2014; Kaewkod et al., 2019; Jakubczyk et al., 2020). Kombucha provides acids (Chen and Liu, 2000; Sreeramulu et al., 2001; Malbaša et al., 2002), sugars (Chen and Liu, 2000), metals, anions, vitamins (Bauer-Petrovska and Petrushevska-Tozi, 2000; Kumar et al., 2008), amino acids, proteins, enzymes, ethanol, antibiotic substances, carbon dioxide, phenol, fiber, tea polyphenols, antioxidants (Malbaša et al., 2011), and trace amounts of alcohol (Jayabalan et al., 2007; Leal et al., 2018). D-saccharic acid-1,4-lactone (DSL) acid, derived from d-glucaric acid, contributes to detoxifying and antioxidant properties (Bhattacharya et al., 2013). The antimicrobial effects of oxalic acid, saccharic acid, gluconic acid, succinic acid, and carbonic acids in kombucha have potential sleep-improving benefits (Sreeramulu et al., 2001). Glucuronic acid and malic acid aid in liver detoxification and limit lipid peroxidation (Jayabalan et al., 2008a). Kombucha fermentation also yields various vitamins, including vitamin B1, known for its anti-aging properties; vitamin B2, which helps prevent arthritis and allergies; vitamin B12, associated with memory enhancement (Bauer-Petrovska and Petrushevska-Tozi, 2000); vitamin B6, which aids in battling depression, stabilizing mood, enhancing concentration, and preventing stroke and obesity; and water-soluble vitamin C (Malbaša et al., 2011), which is known to suppress the release of cortisol (Dufresne and Farnworth, 2000). Kombucha exhibits prophylactic and therapeutic benefits influenced by raw materials, sugar type, fermentation duration, and starter culture (Selvaraj and Gurumurthy, 2023). Demonstrated biological activities include anti-inflammatory (Chakravorty et al., 2016; Vázquez-Cabral et al., 2017), antibacterial (Cardoso et al., 2020), anti-carcinogenic (Jayabalan et al., 2011), antimicrobial (Sreeramulu et al., 2000; Battikh et al., 2012), antioxidant (Chakravorty et al., 2016; Shahbazi et al., 2018; Ivanišová et al., 2020; Jakubczyk et al., 2020), and anti-proliferative effects (Deghrigue et al., 2013; Cardoso et al., 2020). *In vivo* experiments on mice show antioxidative stress effects against chromate (Sai Ram et al., 2000), lead (Dipti et al., 2003), hypoxia, cold (Pauline et al., 2001), and alloxan-induced oxidative stress damage (Aloulou et al., 2012; Bhattacharya et al., 2013). Kombucha prevents myocardial tissue leakage, offering cardiac protection (Lobo and Shenoy, 2015). Anti-diabetic and renoprotective effects against diabetes have been observed (Hosseini et al., 2016; Zubaidah et al., 2019). Kombucha exhibits anti-lipidemic and anti-atherogenic effects against alloxan diabetics (Aloulou et al., 2012; Lobo et al., 2017). Anti-virulence activity against *Vibrio cholerae* (Bhattacharya et al., 2020) and cholesterol-lowering activity (Adriani et al., 2011) have been explored. Kombucha consumption studies on mice demonstrate longevity, improved lifespan, and general health (Hartmann et al., 2000). Kombucha protects against nephrotoxicity by inhibiting lipid peroxidation (Gharib, 2010). Cytogenic activity (Mrdanović et al., 2007) and chromosomal aberrations (Cavusoglu and Guler, 2010) were observed in human lymphocytes. Shenoy confirmed kombucha’s hypoglycemic activity by monitoring blood sugar levels in mice post-consumption (Shenoy, 2000). Banerjee et al. demonstrated kombucha’s ability to heal stomach ulcers in mice (Banerjee et al., 2010). Kombucha aids in halting phenol-induced cytotoxicity (Yapar et al., 2010). Četojević et al. utilized CHO-K1 cell lines from hamsters to investigate protection against genotoxic effects (Četojević-Simin et al., 2012). Kombucha is an easily accessible probiotic drink that can be added to the diet for more nutrition and health benefits. Kim and Adhikari (2020) noted the absence of dedicated work on kombucha’s sensory or consumer evaluation. While some publications mention such research, none offer sufficient or accurate details. Recognizing the growing research interest in kombucha, a study addressing this gap conducted a thorough sensory evaluation and examined consumer preferences. The study utilized suitable methodologies, ensuring an accurate interpretation of the results. It gains attention for its nutrition and high sources of antioxidants from vitamins and polyphenolic compounds. Hence, the nutritional value, antioxidants, and sensory properties of kombucha fermentation from agro-industrial substrates are analyzed.

## 2 Materials and methods

### 2.1 Sample collection

The kombucha starter culture was sourced from Gut Basket through Amazon. Table sugar is the most common choice for a fermentable sugar source. Tea dust was collected from TANTEA-Tamil Nadu Tea Plantation Corporation Limited, Nilgiris, Tamil Nadu (13°02′00.3”N 80°16′03.7″E), which is undertaken by the state government. Molasses was supplied by Vellore Cooperative Sugar Mills and Sugar Research Institute, Vellore, Tamil Nadu (12°58′36.7”N 79°14′25.1″E).

### 2.2 Preparation of kombucha tea

**Figure 1:**
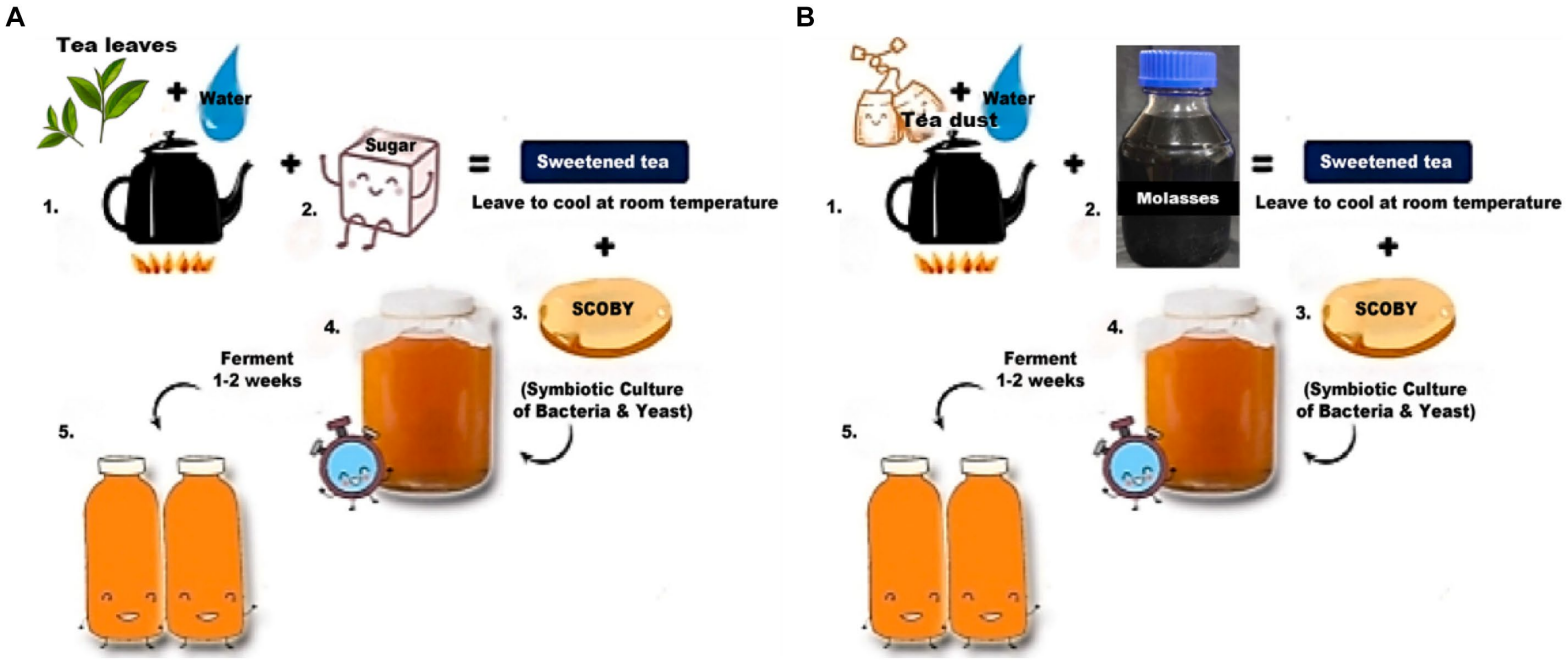
Preparation of kombucha tea control and test samples (Selvaraj and Gurumurthy, 2023). **(A)** Preparation of the control sample. **(B)** Preparation of the test sample.

The control sample had black tea as a nitrogen source and sugar as a carbon source, whereas the test sample had tea dust as a nitrogen source and molasses as a carbon source. The tea leaves and tea dust are from *Camellia sinensis*. Kombucha tea for the control sample was prepared (Figure 1) by boiling 10 g/L of black tea and allowing it to cool to room temperature. 75 g/L of sugar is added to it, and the pH is adjusted to 5 using a digital pH meter by Hanna Instruments (model number Z655295). The test sample was autoclaved at 121°C under 15 psi for 15 min. After cooling, 20 g/L of starter culture was inoculated and left for 10 days of incubation at 30°C (Laavanya et al., 2021). The test sample was prepared (Figure 1) by boiling 10 g/L of tea dust and allowing it to cool to room temperature. 75 g/L of molasses was added to it, and the pH was adjusted to 5 using a digital pH meter by Hanna Instruments (model number Z655295). The test sample was autoclaved at 121°C under 15 psi for 15 min. After cooling, 20 g/L of starter culture (mother) was inoculated into both the test and control samples and left for 6 days of incubation at 30°C (Selvaraj and Gurumurthy, 2023). The mother culture was common for both the control and test samples. The kombucha tea was allowed to ferment for 6 days and collected from control and test samples for metagenomic analysis. The samples are centrifuged and stored at −20°C for further study.

### 2.3 Physicochemical analysis of the raw materials

#### 2.3.1 Physicochemical analysis of the tea leaves and tea dust

The physicochemical analysis of the tea leaves and tea dust was done to determine the differences or similarities in the content of the moisture, total soluble solids, caffeine, polyphenols, nitrogen, and ash in them.

##### 2.3.1.1 Analysis of the moisture content of the tea leaves and tea dust

The moisture content of the tea leaves and tea dust was determined by the International Organization for Standardization (ISO) 7,513. 2 grams of the tea leaves and tea dust samples were taken separately in an aluminium dish and allowed to dry in a hot air oven at 103°C for 4 h. The dried samples are allowed to cool in a desiccator and weighed. The moisture content was calculated using the given formula;


$$\mathrm{Moisture}\;\mathrm{Content}=\left(M_{o}-M_{1}/M_{o}\right)\times 100$$

where M_o_ is the initial weight of the sample and M_1_ is the final weight of the dried sample.

##### 2.3.1.2 Analysis of the total soluble content of the tea leaves and tea dust

The total soluble content in the tea leaf and tea dust samples was estimated by the International Organization for Standardization (ISO) 9,768. The soluble components in the tea leaves and tea dust are extracted by boiling them through reflex. The residues are filtered, washed, and allowed to dry in a hot air oven. The water extract is then determined by weight, and the soluble matter is expressed as a percentage of the mass on a dry basis.

##### 2.3.1.3 Analysis of the caffeine content of the tea leaves and tea dust

The determination of caffeine content in both tea leaf and tea dust samples was conducted using the International Organization for Standardization (ISO) method 10,727, employing High-Performance Liquid Chromatography (HPLC). Caffeine was extracted from the samples through reflux with water in the presence of magnesium oxide. Following filtration, the quantification of caffeine content was performed using high-performance liquid chromatography with an ultraviolet detector, allowing measurements within a wavelength range spanning from 254 nm to 280 nm. A reversed-phase C18 type column was employed, with methanol serving as the mobile phase. After achieving a stable flow rate for the mobile phase and temperature, inject the caffeine standard solutions onto the column, followed by an equivalent volume of the sample solutions. Repeat the injection of standard solutions at regular intervals. Collect and record the data for the peaks observed in both the standards and test samples.

##### 2.3.1.4 Analysis of the nitrogen content of the tea leaves and tea dust

The determination of total nitrogen content in tea leaf and tea dust samples followed the International Organization for Standardization (ISO) method 16,634. The Dumas method, an analytical technique for quantifying total nitrogen content, involves a process of combustion, reduction, separation, and detection. In this method, tea samples are heated to high temperatures and burned in the presence of oxygen, producing carbon dioxide, water, and nitrogen oxides. The gas mixture undergoes reduction and separation over hot copper to eliminate oxygen and convert nitrogen oxides to nitrogen. Traps are employed to remove water and carbon dioxide from the sample. The released total nitrogen content was measured and expressed as a percentage mass fraction in the results.

##### 2.3.1.5 Analysis of the polyphenol content of the tea leaves and tea dust

The evaluation of polyphenol content in tea leaf and tea dust samples adhered to the standards outlined in the International Organization for Standardization (ISO) 14,502-1, utilizing the Folin–Ciocalteu colorimetric method. The analytical tools employed included an analytical balance, a water bath, pipettes, tubes, flasks, and a spectrophotometer. The key reagents involved in the process were acetonitrile, methanol, sodium carbonate solution, gallic acid, and Folin–Ciocalteu phenol reagents. The test and control samples were stabilized by dissolving them in hot water with 10% acetonitrile. Then Folin–Ciocalteu reagent, followed by sodium carbonate, was added to 10 mL of the filtered control and test samples. The mixture was then incubated for two hours at room temperature (37°C). The phospho-tungstic acids in the samples are oxidized to phenolic hydroxy groups by the Folin–Ciocalteu reagent, and a blue colour is produced. The absorbance was measured at 765 nm on a BioTek 800 TS Absorbance Reader (model number 40-006) with ethanol as the blank. The polyphenolic content of the samples was determined with gallic acid as a reference standard. Total polyphenol was reported as gallic acid equivalent (GAE)/g of the sample.

##### 2.3.1.6 Analysis of the ash content of the tea leaves and tea dust

The evaluation of ash content in tea leaf and tea dust samples adhered to the standards outlined in the International Organization for Standardization (ISO) 1,575. The samples were taken in a dish and heated at 525 ± 25°C in a muffle furnace until the ash was visibly free from carbon particles. The ash was cooled, moistened with distilled water, dried in a steam bath, and then placed on a hot plate. The sample was taken in the dish, returned to the furnace, heated for 60 min, cooled in a desiccator, and weighed. Subsequently, it underwent another heating cycle in the furnace for 30 min, followed by cooling and weighing. This multi-step process is crucial for the accurate determination of the ash content in the tea leaves and tea dust.

#### 2.3.2 Physicochemical analysis of molasses

The physicochemical analysis of the molasses was done to determine the pH, brix, specific gravity, and total sugars in them.

##### 2.3.2.1 Analysis of the pH of molasses

A digital pH meter (Hanna Advanced pH Benchtop Meter-HI6221-02) was used to measure the pH of the molasses sample by the ISO 10523 method. The pH estimation was done to determine the acidity or alkalinity by measuring the concentration of hydrogen ions in the molasses.

##### 2.3.2.2 Analysis of the brix of molasses

A Labart (0–90% brix model) handheld refractometer was used to determine the brix value of the molasses. It was used to measure the sugar (sucrose) concentration in the molasses by weight by the ISO 2173 method. The refractometer measures the refraction of light as it passes through a liquid. The greater the concentration of sugar in the liquid, the higher the degree of light refraction.

##### 2.3.2.3 Analysis of the specific gravity of molasses

The evaluation of specific gravity content in molasses was conducted following the guidelines outlined in the International Organization for Standardization (ISO) 10,349-11. A volume of 2 mL of molasses was carefully placed in the test cylinder and stabilized at 20°C to ensure accurate readings. The insertion of a hydrometer into the sample provided a quantitative measure of the relative density of molasses when compared to the standard density of water.

##### 2.3.2.4 Analysis of the sugars in molasses

The sugars (fructose, glucose, and sucrose) in the samples were analyzed using high-performance liquid chromatography (HPLC) by following the method from Wan (Wan Sapawi and Hussin, 2019). The sugars were quantified based on calibration curves compared to the maltose as standard. 6 μL of filtered control and test samples of the kombucha tea were injected into a HPLC system equipped with a refractive index detector by an isocratic pump and a column. A mixture of acetonitrile and deionised water was used as a mobile phase. The flow rate of 1 mL/min was maintained at room temperature. The retention times of each sugar are recorded as resolution peaks at 220 nm. The concentrations of the sugars in both the control and test samples were quantified with reference to the standard curves (Wan Sapawi and Hussin, 2019).

##### 2.3.2.5 Analysis of the nitrogen content in molasses

The determination of total nitrogen content in molasses sample followed the International Organization for Standardization (ISO) method 16,634. The Dumas method, an analytical technique for quantifying total nitrogen content, involves a process of combustion, reduction, separation, and detection. In this method, the molasses was heated to high temperatures and burned in the presence of oxygen, producing carbon dioxide, water, and nitrogen oxides. The gas mixture undergoes reduction and separation over hot copper to eliminate oxygen and convert nitrogen oxides to nitrogen. Traps are employed to remove water and carbon dioxide from the sample. The released total nitrogen content was measured and expressed as a percentage mass fraction in the results.

### 2.4 Microbial diversity of kombucha

The microbial diversity analysis of kombucha followed the protocol established by Kaashyap and Pradhan, as outlined in their studies (Kaashyap et al., 2021; Pradhan et al., 2023). 1 mL of 6 day fermented kombucha tea was filtered and collected from the control and test samples. 1 gram of cellulosic fermented (SCOBY) from the control and test samples was collected and chopped into small fragments using a sterile blade. The tea and SCOBY samples were collected in sterile microfuge tubes to extract the DNA and explore the microbial diversity. The whole genome sequencing of the SCOBY layer of the control and the test was analyzed by 16S and internal transcribed sequencing (ITS). 16S and ITS sequencing are commonly used amplicon sequencing methods to identify the bacteria and fungi present in a sample. The 16S sequencing method targets the 16S rRNA gene, whereas the ITS sequencing method targets the internal transcribed spacer regions of fungal DNA. The DNA extraction was done using the commercially available gDNA xplorogen kit as per the manufacturer’s recommendation. The kombucha samples K1 and K2 are added to the microfuge tube containing lysis buffer and proteinase K. Vortex thoroughly until a homogeneous slurry is obtained, and incubate at 65°C for 15 min for cell lysis. An equal volume of phenol, chloroform, and isoamyl alcohol was added to the lysate and mixed gently. Then centrifuge at 10,000 rpm for 5 min to separate the supernatant and pellet. Transfer the supernatant to a new tube and add isopropanol to the aqueous phase to precipitate DNA. Mix the solution and incubate at −20°C for at least 30 min. Centrifuge at 10,000 rpm (4°C) for 10 min to pellet the DNA. Wash the DNA pellet with 70% ethanol to remove impurities. Centrifuge at 10,000 rpm for 5 min and carefully remove the ethanol. The DNA pellet was allowed to air-dry to remove any remaining ethanol. The purified DNA was resuspended in sterile, distilled water. The V3-V4 region of the 16 s gene was amplified by PCR. The TAQ master mix was composed of high-fidelity DNA polymerase, 0.5 mM dNTPs, 3.2 mM MgCl_2_, and PCR enzyme buffer. 40 nanograms of the extracted DNA are used for amplification, along with 10 pM of each primer. The primers used for 16 s were 16sF-5’ AGAGTTTGATGMTGGCTCAG3’ and 16sR-5’ TTACCGCGGCMGCSGGCAC3’. The primers used for ITS sequencing were ITS1-5’ TTGGTCATTAGAGGAAGTAA 3′ and ITS2-5’ GCTGCGTTCTTCATCGATGC3’. The PCR conditions for 25 cycles were systemized. The initial denaturation was at 95°C, followed by denaturation at 95°C for 15 s, annealing at 60°C for 15 s, and elongation at 72°C for 2 min. The final extension was at 72°C for 10 min and halted at 4°C. The amplified DNA can then be further analyzed or sequenced for downstream applications, such as identifying microbial communities in the case of 16S gene amplification or characterizing fungal communities with ITS sequencing. The extracted DNA was purified and subjected to library preparation. The amplicons from the control and the test samples were purified with ampure beads. It removed the unused primers, leaving only the desired DNA fragments. Additionally, 8 cycles of PCR were performed using Illumina barcoded adapters, which involve attaching sequencing adapters with unique barcodes to the purified amplicons. These barcodes allow for the identification of individual samples during the sequencing process. Libraries were purified using Ampure beads to retain only fragments with attached adapters for sequencing. Quantification was performed with the Qubit dsDNA High Sensitivity assay kit. Sequencing utilized the Illumina Miseq 2x300PE v3 kit for accurate quantification of double-stranded DNA with 300 base pair reads. Raw data underwent quality checks using FASTQC and MULTIQC. TRIM GALORE trimmed adapters and low-quality reads, enhancing sequencing data quality. Trimmed reads were merged, and chimeric sequences were removed during PCR. QIIME facilitated microbial community analysis, including operational taxonomic unit (OTU) clustering, while KRAKEN provided taxonomic classification. The workflow involved abundance calculation, error correction, and accurate investigations at the genus level. Each read was classified based on coverage (%) and identity, offering taxonomic insights at the genus level. This 16S workflow is valuable for identifying pathogens in mixed samples and understanding microbial community composition. Accurate investigations at the genus level provide insights into microbial diversity and abundance.

### 2.5 Biochemical analysis and antioxidant activity of kombucha

The concentrations of lactic acid, acetic acid, citric acid, gluconic acid, and glucuronic acid in both the control and test samples were determined by high-performance liquid chromatography (HPLC) (Jayabalan et al., 2007). Analytical balance, filtration unit, membrane filter, pipettes, tubes, flasks, diode array detector, Phenomenex Luna C-18 (2) column, and HPLC. Potassium dihydrogen phosphate, methanol, and standards for lactic acid, acetic acid, citric acid, gluconic acid, and glucuronic acid. The organic acid content in the control (black tea and sugar) and the test (tea dust and molasses) was observed for 12 days of fermentation with an interval of 3 days. 5 mL of the filtered control sample and test sample were analyzed for lactic acid, acetic acid, citric acid, gluconic acid, and glucuronic acid by high-performance liquid chromatography (HPLC). A 10 μL sample was injected into an HPLC system with a diode array detector and a Phenomenex Luna C-18(2) column. The potassium dihydrogen phosphate and methanol mixture (97:3) was used as the mobile phase. The flow rate of 1 mL/min was maintained at 28°C. The retention times of each acid are recorded as resolution peaks at 220 nm. The concentrations of organic acids in both the control and test samples were quantified with reference to the standard curves (Jayabalan et al., 2007). The tannin content in the kombucha tea samples was analyzed by BioTek 800 TS Absorbance Reader (model number 40-006) at a wavelength of 720 nm (Muzaifa et al., 2023). The flavonoids in the control and test samples were measured at an absorbance of 510 nm. It was determined using rutin as a reference standard for the calibration curve. The analysis was performed in triplicate (Jakubczyk et al., 2020). The total polyphenol content was determined using the Folin–Ciocalteu colorimetric method (ISO 14502-1). Folin–Ciocalteu reagent, followed by sodium carbonate, was added to 10 mL of the filtered control and test samples. The mixture was then incubated for 2 hours at room temperature (37°C). The absorbance was measured at 750 nm on the BioTek 800 TS Absorbance Reader (model number 40-006) with ethanol as the blank. The polyphenolic content of the samples was determined with gallic acid as a reference standard. Total polyphenol was reported as gallic acid equivalent (GAE)/g of the sample. The analysis was carried out in triplicate (Chakravorty et al., 2016). 0.3 mL of ferric chloride and potassium ferricyanide were added to 1 mL of the control and test samples, shaken, and allowed to settle. The antioxidant activity of the control (black tea and sugar) and test (tea dust and molasses) was observed for 12 days of fermentation by FRAP and DPPH assays. A ferric ion reducing antioxidant power (FRAP) assay was conducted to assess the reducing capability of antioxidants in the kombucha tea samples (Benzie and Strain, 1996). During this process, 100 μL of kombucha was mixed with 700 μL of the FRAP working solution. The FRAP working solution was prepared by combining a solution of 0.3 M acetate buffer, 10 mM TPTZ in 40 mM HCl, and 20 mM ferric chloride in a ratio of 10:1:1. After a 60 min incubation in darkness, the absorbance at 593 nm was measured using a microplate reader. The FRAP antioxidant activity was determined by comparing it to a calibration curve, with ferrous sulfate utilized as the standard and expressed as mmol Fe (II)/mL. The 2,2-diphenyl-1-picrylhydrazyl (DPPH) technique was used to measure antioxidant activity in studies due to its stability. The graphs were plotted using Microsoft Excel and GraphPad Prism 9.5.0 software. 100 μL of the test sample was mixed with 1 mL of 0.1 mM DPPH in ethanol and 450 μL of 50 mM Tris-HCl buffer with a pH of 7.4. The solution was incubated in the dark for 30 min at room temperature. The free-radical reduction was measured using an ELISA plate reader at 517 nm. A sample with black tea and sugar was taken as a control, whereas a sample with tea dust and molasses was taken as a test (Jayabalan et al., 2008b). This activity is given as % DPPH radical scavenging, calculated according to the following equation:


$$\begin{array}{l} \mathrm{DPPH}\;\mathrm{radical}\;\\ \mathrm{scavenging}\;\mathrm{activity}\;\left(\%\right)=\left[\begin{array}{l} \left(\begin{array}{l} \mathrm{control}\;\mathrm{absorbance}-\\ \mathrm{sample}\;\mathrm{absorbance} \end{array}\right)/\\ \mathrm{control}\;\mathrm{absorbance} \end{array}\right]\times 100 \end{array}$$

### 2.6 Nutritional profiling of kombucha tea

Nutritional analysis was done to estimate the nutrition content of the fermented tea from control and test samples. Black tea with sugar as a substrate for kombucha beverages was used as a control sample. A black tea made from tea dust and blackstrap molasses was used as a test sample. Kombucha has high beneficial values and is taken as a refreshment beverage (Sreeramulu et al., 2000). The nutritional content of kombucha tea is analyzed using sophisticated instruments. The experiments were done in triplicate, and the values were noted. This contributes to the robustness and reliability of the results.

#### 2.6.1 Estimation of sugars in kombucha tea

The sugars (fructose, glucose, and sucrose) in the samples were analyzed using high-performance liquid chromatography (HPLC) by following the method from Wan (Wan Sapawi and Hussin, 2019). The sugars were quantified based on calibration curves compared to the standard. Analytical balance, filtration unit, membrane filter, pipettes, tubes, flasks, isocratic pump, refractive index detector, and HPLC. Standards of fructose, glucose, sucrose, acetonitrile, and deionised water. 6 μL of filtered control and test samples of the kombucha tea was injected into a HPLC system equipped with a detector by an isocratic pump and a column. A mixture of acetonitrile and deionised water was used as a mobile phase. The flow rate of 1 mL/min was maintained at room temperature. The retention times of each sugar are recorded as resolution peaks at 220 nm. The concentrations of the sugars in both the control and test samples were quantified with reference to the standard curves (Wan Sapawi and Hussin, 2019).

#### 2.6.2 Estimation of minerals in kombucha tea

Kombucha tea has trace elements of minerals that provide nutritional significance. The samples were analyzed according to the Food Safety and Standards Authority of India (FSSAI) manual of methods of food analysis by atomic absorption spectrophotometer (2016). Analytical balance, filtration unit, membrane filter, pipettes, tubes, flasks, and atomic absorption spectrophotometer. Standard solutions of minerals such as calcium, iron, copper, and potassium are used as references. The control and test samples were analyzed for minerals such as calcium, iron, copper, and potassium by an atomic absorption spectrophotometer. The air acetylene flame induces the ionization of the minerals that are detected by the spectrometry. Potassium and calcium are measured at a wavelength of 766.5 nm and 422.7 nm, respectively. A calibration graph was plotted with absorbance readings against the mass concentrations of each mineral in the samples.

#### 2.6.3 Estimation of vitamins in kombucha tea

The concentrations of vitamins in both the control and test samples were determined by high-performance liquid chromatography (HPLC) (Jayabalan et al., 2007). Analytical balance, filtration unit, membrane filter, pipettes, tubes, flasks, detector, column, and HPLC. Standard solutions of vitamins (B1, B2, B3, B6, B9, B12, and C) for reference. The vitamin (B1, B2, B3, B6, B9, B12, and C) content in the control (black tea and sugar) and the test (tea dust and molasses) was observed for 12 days of fermentation with an interval of 3 days. 5 μL of filtered control and test samples of the kombucha tea was injected into a HPLC system equipped with a detector by an isocratic pump and a column. The flow rate of 1 mL/min was maintained at room temperature. The retention times of each vitamin are recorded as resolution peaks at 270 nm. The concentrations of the vitamins in both the control and test samples were quantified with reference to the standard curves (Jayabalan et al., 2007).

#### 2.6.4 Estimation of ethanol content in kombucha tea

The ethanol content in the samples was determined by the dichromate method and quantified using the BioTek 800 TS Absorbance Reader (model number 40-006) based on the retention time (Muzaifa et al., 2023). An analytical balance, pipettes, test tubes, beakers, a cuvette, and a spectrophotometer are required for the experiment. Potassium dichromate, sulfuric acid, potassium sulphate, distilled water, and ethanol. 1 mL of potassium dichromate, sulfuric acid, and potassium sulphate were mixed with 2 mL of the control and the test samples, respectively. The samples were incubated in the dark for 2 h. A green colour is formed if the sample contains ethanol in it. 1 mL of the sample is diluted with 25 mL of distilled water and measured at 460 nm using the BioTek 800 TS Absorbance Reader (model number 40-006) (Wan Sapawi and Hussin, 2019).

#### 2.6.5 Estimation of protein content in kombucha tea

The total protein concentration in the control and test samples was quantified by the Lowry method colorimetric assay (Lowry et al., 1951). The proteins in the samples reacted with the Folin–Ciocalteu reagent and produced a blue coloured phosphomolybdotungstate complex. The intensity of the color is directly proportional to the concentration of proteins in the sample. An analytical balance, filtration unit, membrane filter, pipettes, test tubes, beakers, water bath, hot air oven, a cuvette, and the BioTek 800 TS Absorbance Reader (model number 40-006) required for the experiment. The reagent A is an alkaline copper solution, which is made of 2% sodium carbonate (Na₂CO₃) solution and 1% copper sulfate (CuSO₄) solution. The reagent B is the Folin–Ciocalteu phenol reagent, which is commercially available. Bovine serum albumin (BSA) is used as the protein standard. A standard curve is created by a series of 0, 25, 50, 100, and 200 μg/mL of known protein concentration with bovine serum albumin (BSA) as a protein standard. The sample concentration was within the range of the standard curve. Reaction tubes were set for standards, samples, and blanks. 0.1 mL of the protein standard and 2.0 mL of Reagent A (alkaline copper solution) were added to the sample and mixed well. The tubes were incubated at room temperature for 10 min. After the incubation, 0.2 mL of Reagent B (Folin–Ciocalteu reagent) was added to each tube and mixed thoroughly. The tubes were incubated at room temperature for 30 min. The absorbance of each tube was measured at 750 nm using the BioTek 800 TS Absorbance Reader (model number 40-006). The development of the blue colour was observed. The blank with the reagents was used for reference. The absorbance values of the standard solutions were plotted against their respective concentrations to create a standard curve. The standard curve was used to determine the protein concentration of the samples based on the absorbance values (Lowry et al., 1951).

#### 2.6.6 Estimation of total fat in kombucha tea

The total fat content was determined by using a solvent extraction method (ISO 11085) that involves extracting fat using a suitable petroleum ether in a Randall-type apparatus. The extracted fat in the kombucha tea sample was then determined gravimetrically. An analytical balance, filtration unit, membrane filter, pipettes, tubes, beakers, a water bath, and a hot air oven are required for the experiment. A drying oven is used for the drying of the fat from the sample. An analytical balance is used to measure the extracted fat content. Randall extraction apparatus is generally used for determining fat content. Petroleum ether was used as a solvent for the extraction. 5 g of test and control samples were loaded into the Randall extraction apparatus. Petroleum ether was added and left for 15 h to extract the fat from the sample. The extract was filtered and transferred to a conical flask, and the solvent was allowed to evaporate. The sample in the flask was dried in an oven at 80°C. The extracted fat obtained was weighed to determine the fat content of the control and test samples, respectively.

#### 2.6.7 Estimation of fiber in kombucha tea

The fiber content in the test and control samples of kombucha tea was quantified by (ISO 15598) International Standard Guidelines. The tea was diluted with an acid and alkali solution to remove soluble substances where the fiber was settled. An analytical balance, crucible, filtration unit, membrane filter, pipettes, tubes, beakers, water bath, digestion flasks, muslin cloth, and a hot air oven are required for the experiment. The reagents, like sulphuric acid (acid) and sodium hydroxide (alkali) solution, are required for digestion, washing, and neutralization. 2 g of the test and samples were transferred into separate beakers. 200 mL of boiling sulphuric acid was added to each beaker. It was connected to the digestion apparatus and boiled for 30 min. The samples were filtered through muslin cloth, and the leftover residue on the cloth was transferred into the flask with 200 mL of boiling sodium hydroxide solution. The flask was connected immediately to the digestion apparatus and boiled for 30 min. The flask was removed and then filtered by the Gooch crucible. It was washed with hot water until it was free from alkali, and then with 10 mL of alcohol. The samples were dried at 100°C in a hot air oven for 2 h, allowed to cool, and weighed. The crude fiber in the samples was determined by the weight of the residue.

#### 2.6.8 Estimation of sodium in kombucha tea

ISO 9964-2 specifies a method for determining the dissolved sodium in the samples using flame atomic absorption spectrometry. The sodium in the sample is atomized by a flame furnace, and the concentration is measured at an absorbance of 590 nm. An analytical balance, filtration unit, pipettes, membrane filter, conical flasks, water bath, test tubes, a cuvette, and flame atomic absorption spectrometry are required for the experiment. An atomic absorption spectrometer is used for wavelength measurement. Hydrochloric acid (HCl), nitric acid (HNO_3_), caesium chloride (CsCl), distilled water, and sodium solution. The caesium chloride solution is used as an ionization suppressant. The control and test samples of kombucha tea were filtered using a 0.45 μm pore size filter. 2 mL of the samples were transferred to a 100 mL conical flask, and 10 mL of the caesium chloride solution was added to each of them. The concentration of the samples was adjusted to the optimum range of 0.l mg/l to 1.0 mg/L of sodium using distilled water. Add 10 mL of caesium chloride solution to each of a series of 100 mL one-mark volumetric flasks to make the calibration solution. Pipette out 0 mL, 1 mL, 2 mL, 4 mL, 6 mL, and 10 mL of the sodium standard solution and make up the calibration solution concentrations as 0 mg/L, 0.l mg/l, 0.2 mg/L, 0.4 mg/L, 0.6 mg/L, and 1.0 mg/L of sodium using distilled water. The samples were observed at 590 nm, and distilled water was used as a blank. A calibration graph was plotted with absorbance readings against the mass concentrations of sodium in the samples.

#### 2.6.9 Estimation of carbohydrate content in kombucha tea

The carbohydrate content in the control and test samples was determined by ISO 11292, an international standard, using high-performance anion-exchange chromatography. It involves the separation of carbohydrate components from the filtered samples by ion chromatography on a high-performance anion-exchange chromatography, followed by detection and quantification. Sodium hydroxide, demineralized water (eluent 1), hydrochloric acid (eluent 2), and carbohydrate standard (arabinose, fructose, galactose, glucose, mannose, sucrose, and mannitol). 100 mg of each carbohydrate are diluted to make up 1,000 mg/L in separate flasks. Analytical balance, volumetric flask and cylinders, vacuum filtration system, membrane filter, water bath, disposable membrane filters and C18 filter cartridges, metal-free filter chromograph, integrator, and pulsed amperometric detector (PAD). The standard and test solutions were filtered through 0.2 μm membrane filters. 20 μL of filtered standard and test solutions were injected into the chromatograph, and the carbohydrates were separated. The column flowrate was 1 mL/min, and the post-column flowrate was 0.6 mL/min with eluent 2 at ambient temperature. The carbohydrates in the sample solution were identified, quantified, and compared with retention times and peaks obtained with reference to the standard solution.

The carbohydrate content (*ω*), expressed as a percentage by mass, is equal to,


$$\omega =A-m_{0}-V\times 100$$

$$A_{0}-m-V_{0}$$

*A* is the peak area of the individual carbohydrate in the test. *A*_0_ is the peak of the individual carbohydrate in the standard. *m* is the mass of the test, and *m*_0_ is the mass of the carbohydrate in the standard. *V* is the volume of the test, and *V*_0_ is the volume of the standard.

#### 2.6.10 Estimation of calories in kombucha tea

The Atwater system was generally used to determine the energy value based on the contribution of protein, fat, carbohydrates, and alcohol components of the sample. Energy was calculated by using the general Atwater factor of 4 kilocalories (kcal) per gram of protein, 9 kcal per gram of fat, and 4 kcal per gram of carbohydrate (Merrill, Usda Handbook 74, 1995).


$$\begin{array}{l} \mathrm{Energy}\;\left(\mathrm{kcal}\right)=\left(4\;\mathrm{kcal}/g\times g\;\mathrm{protein}\right)+\\ \;\left(4\;\mathrm{kcal}/g\times g\;\mathrm{carbohydrate}\right)+\\ \;\left(9\;\mathrm{kcal}/g\times g\;\mathrm{fat}\right)\text{.} \end{array}$$

#### 2.6.11 Estimation of cholesterol in kombucha tea

The cholesterol content in the control and test samples was determined by ISO 11702, an international standard determined enzymatically using the BioTek 800 TS Absorbance Reader (model number 40-006). Cholesterol standard solution, chloroform, enzymes such as cholesterol esterase and cholesterol oxidase, hydrogen peroxide, test tubes, flasks, pipettes, a cuvette, and a spectrophotometer. The cholesterol in the control and test samples was extracted using chloroform. The cholesterol esterase enzyme has hydrolyzed the cholesterol esters into cholesterol. Then the free cholesterol is oxidized to cholest-4-en-3-one and hydrogen peroxide by cholesterol oxidase. The hydrogen peroxide was reacted to form yellow-coloured 4-aminoantipyrine and phenol in the presence of the peroxidase enzyme. The coloured solution was further measured at 550 nm using the BioTek 800 TS Absorbance Reader (model number 40-006). A calibration curve was constructed using known concentrations of the cholesterol standard solution. The concentration of cholesterol in the sample was determined based on the absorbance and the calibration curve.

#### 2.6.12 Estimation of caffeine in kombucha tea

The determination of caffeine content in both tea leaf and tea dust samples was conducted using the International Organization for Standardization (ISO) method 10,727, employing High-Performance Liquid Chromatography (HPLC). Caffeine was extracted from the samples through reflux with water in the presence of magnesium oxide. Following filtration, the quantification of caffeine content was performed using high-performance liquid chromatography with an ultraviolet detector, allowing measurements within a wavelength range spanning from 254 nm to 280 nm. A reversed-phase C18 type column was employed, with methanol serving as the mobile phase. After achieving a stable flow rate for the mobile phase and temperature, inject the caffeine standard solutions onto the column, followed by an equivalent volume of the sample solutions. Repeat the injection of standard solutions at regular intervals. Collect and record the data for the peaks observed in both the standards and test samples.

### 2.7 Sensory analysis

#### 2.7.1 Membrane filtration and coliform test

A filtration column was connected to a vacuum pump and used for membrane filtration. A nitrocellulose membrane with a pore size of 0.45 μm is used for filtration to capture bacterial cells present in a sample. A nitrocellulose membrane is placed between the chamber and catchment vessels. When the vacuum is passed, the sample gets filtered through the nitrocellulose membrane and collected in the vessel. 100 mL of each sample, the test and the control, were filtered separately (Forster and Arango Pinedo, 2016). A coliform test is performed to test the quality of the sample and detect pathogenic fecal coliforms in the sample. Eosin-Methylene Blue (EMB) agar is generally used in coliform testing. The kombucha tea made from black tea and sugar was taken as the control, and the tea made from tea dust and molasses was taken as the test. Both the tea samples were subjected to a coliform test to check their quality. 50 mL of EMB agar was prepared and sterilized at 121°C for 15 min. The plates were poured and labelled as control and test. The membrane filter with residues from the control and test samples was aseptically removed using sterile forceps and placed on the agar plate. The plates were labelled and allowed to incubate at 35°C for 24 h (Abu-Sini et al., 2023). After incubation, the plates were examined for any bacterial coliform colonies. The results were used to evaluate the quality of the kombucha tea samples to ensure their safety and identify contamination that poses a health risk to consumers.

#### 2.7.2 Evaluation of organoleptic properties

Sample A is the control sample, whereas sample B is the test. Production of 1 litre of samples A and B included 20 grams of SCOBY (Symbiotic Culture of Bacteria and Yeast) and 10 mL of starter tea in 1 litre of water. Sample A had 10 grams of black tea powder as a nitrogen source and 75 grams of sugar as a carbon source, whereas sample B had 10 grams of tea dust as a nitrogen source and 75 grams of molasses as a carbon source. The starter tea refers to the fermented tea, whereas the SCOBY layer is the cellulose layer that is formed in the air-liquid interface from the previous batch of fermentation (Villarreal-Soto et al., 2018). The ingredients and proportions of samples A (control) and B (test) of fermented tea for sensory analysis were prepared according to Table 1.

**Table 1:** Ingredients and proportions used for the production of the fermented tea.

Sample A (Control)		Sample B (Test)	
Ingredients	Quantity/L	Ingredients	Quantity/L
SCOBY	20 g	SCOBY	20 g
Starter tea	10 mL	Starter tea	10 mL
Black tea powder	10 g	Tea dust	10 g
Water	1 L	Water	1 L
Sugar	75 g	Molasses	75 g

The panel members were asked to answer five questions for both the test and control samples. And also, they were asked their preference between the test sample and the control sample. The five questions include the smell, oral texture, colour, taste, and overall acceptability. All five questions were given four options to choose from. The smell options are comprised of fruity, citrus, pleasant, and stinky. The oral texture options are comprised of soft, fizzy, creamy, and sticky. The colour options were comprised of bright, pale, average, and bad. The taste options are comprised of acidic, fruity, alcoholic, and sweet. Lastly, respondents assessed overall acceptability with options such as excellent, good, average, and poor. All these questions had a criterion to understand the acceptability of the potential health drink on the market. A consent form was given to the panel members along with the questionnaire and a note. It stated that they volunteered and were convinced by the organizer to try at their own risk. They were requested to take the sensory tests and give an honest review to share their genuine perceptions. The evaluation of the organoleptic characteristics of the kombucha tea beverage was conducted through the participation of an untrained taste panel consisting of 100 evaluators within the age range of 25 to 35 years. They were asked to give a hedonic rating by evaluating key attributes including smell, appearance, flavor, taste, and overall acceptability. A comparative analysis was carried out on all the parameters between the control sample A (black tea and sucrose) and the test sample B (tea from tea dust and molasses).

### 2.8 Statistical analysis

The results were expressed as mean ± standard deviation. The differences between the means were analyzed by a student’s *t*-test at the *p* < 0.05 level. The results were statistically analyzed by Microsoft Excel and GraphPad Prism 9.5.0 software.

## 3 Results and discussion

### 3.1 Physicochemical analysis of the raw materials

Traditionally kombucha is made from black tea and sugar as substrates (Selvaraj and Gurumurthy, 2023). We propose to use cheaper by-products that is tea dust and blackstrap molasses as nitrogen and carbon source for kombucha production. Hence these raw materials were analyzed for physico-chemical characters.

#### 3.1.1 Physicochemical analysis of the tea leaves and tea dust

Tea leaves are harvested from the *Camellia sinensis* plantation, whereas tea dust is spilled waste material that is obtained during the conversion of the leaves into dried leaves (Khan et al., 2016). The physicochemical properties of the leaves and dust have a slighter difference due to various factors during the processing method. Moisture content is an important parameter of tea for quality assurance in the tea industry. Tea is generally hygroscopic, which means it observes the moisture content in the air easily, which leads to contamination and deterioration in the brewing quality. The shelf life, chemical, and sensory properties should be maintained by proper storage to protect against moisture content (Aaqil et al., 2023). The moisture content in the tea dust (3.5 ± 0.3%) was higher than the tea leaves (3.1 ± 0.5%) due to the increased surface area of the tea dust, which could potentially cause moisture absorption from the air (Shchegoleva et al., 2021). The ISO 9768 standard suggested that the moisture content of the tea sample should be in the range of 3–7% for a good storage condition. Since the moisture content of both tea leaves and tea dust are similar the substitution may not affect the final process. Tea leaves contain a minimum of 25% solid matter that is soluble in water, referred to as extract. However, the quantity of extracts tends to decrease as the intervals between plucking lengthen and as the leaves undergo aging. The tea’s aqueous extract should be at least 32% of its dry mass, as specified by ISO 3720. The soluble matter in the tea leaves was 35.3 ± 0.3% and the tea dust was 43.2 ± 0.4%, which implies that the tea leaves have higher soluble content like polyphenols, caffeine, acids, and minerals (Shchegoleva et al., 2021). The tea leaf had a higher caffeine value of 1.8 ± 0.4%, and the tea dust had 2.1 ± 0.2%. The presence of tea stems in tea dust, along with the cut, crushed, or torn processing, increases the surface area of the tea particles, promoting a higher extraction of caffeine during brewing. The tea leaves have less exposed surface area, resulting in a less concentrated brew and a lower caffeine level (Aroyeun, 2012). In the context of kombucha fermentation, the caffeine found in tea infusion serves as a nitrogen source for the synthesis of bacterial cellulose during the fermentation process (Jakubczyk et al., 2020). The increased caffeine content in tea dust might help to increase the kombucha production. Tea contains essential macronutrients such as nitrogen, phosphorus, and potassium, contributing to its nutritional value. The nitrogen content serves as a crucial indicator for assessing tea quality, with good-quality tea typically exhibiting a nitrogen content ranging from 3 to 7%. Tea leaves, with a nitrogen content of 4.9 ± 0.3%, have a higher nitrogen concentration compared to tea dust, which has a nitrogen content of 4.4 ± 0.2%. This difference is attributed to the processing method, which results in a loss of nitrogen content, favouring higher concentrations in tea leaves over tea dust (Shchegoleva et al., 2021). According to the International Organization for Standardization (ISO) 11,287, the polyphenol content in the tea sample should range from 15–35%. The polyphenol content in tea leaf was 17.5 ± 0.4%, whereas the tea dust was 23.8 ± 0.5% higher due to the high catechin content. The reduction in polyphenol levels in tea leaves results from the oxidative fermentation of catechins during the primary processing of fresh leaves (Shchegoleva et al., 2021). The catechin in the dust is high due to the presence of broken stems along with the tea leaves. Both the leaf and the dust have rich antioxidant content that protects against free radicals. According to the International Organization for Standardization (ISO) 1,575, the ash content should fall within a range of approximately 4 to 7.5% on a dry weight basis for an overall good quality of the tea. The total ash content in the tea dust was 6.3 ± 0.3% and in the tea leaves was 5.7 ± 0.2%. The higher ash content in tea dust suggests a richer mineral composition in comparison to tea leaves (Shchegoleva et al., 2021).

#### 3.1.2 Physicochemical analysis of molasses

Sucrose is a common ingredient in fermentation processes because it provides a readily available source of fermentable sugars that microorganisms can metabolize. In the context of kombucha fermentation, sugar is a critical component as it is the primary energy source for the SCOBY (Symbiotic Culture of Bacteria and Yeast). Molasses, being rich in sucrose and other nutrients, can be used as a substitute for traditional sugar in kombucha fermentation. We found that the sucrose content was 38.2 ± 0.3% in the blackstrap molasses. Even though the sucrose content is lower, the presence of glucose (6.3 ± 0.5%) and fructose (10.8 ± 0.2%) helps the kombucha fermentation. This analysis provides valuable information about various aspects of the molasses, including its pH, brix, specific gravity, and sugar. The utilization of blackstrap molasses in this process has the additional benefit of introducing unique flavors and nutrients to the final product, thereby contributing to a distinct and intricate profile that influences the taste and characteristics of the kombucha (Devanthi et al., 2021). Blackstrap molasses is a byproduct of sugar refining, and its pH is influenced by the presence of organic acids formed during the sugar extraction process. The pH of blackstrap molasses is typically in the range of 4.5 to 5.5, making it slightly acidic. The pH of the blackstrap molasses was 5.3 ± 0.1, which was suitable for the activity of yeast during fermentation (Devanthi et al., 2021). The Brix value serves as a percentage measure for the total soluble solids in a solution, including sugars, organic acids, salts, and proteins. The brix value is often used as an indicator of the overall sweetness or concentration of a solution. The brix value of the blackstrap molasses was determined to be 76.4 ± 0.2% using the handheld refractometer. This falls within the standard range of 75 to 85%, affirming that blackstrap molasses is a notably rich source of sugars and nutrients, as highlighted in the study by Devanthi et al. (2021). The molasses had a good source of total nitrogen content 1.3 ± 0.2% which also enhanced the yeast growth in the culture during the fermentation process (Devanthi et al., 2021). The specific gravity of molasses, measured at 1.4 ± 0.1, aligns with findings from Devanti’s study and falls within the standard range of 1.35–1.45% (Devanthi et al., 2021). The specific gravity provides valuable information about the concentration, composition, and characteristics of molasses to maintain quality, optimize processes, monitor fermentation, and ensure the desired outcome. The sugar composition of molasses is detailed with specific percentages, indicating the presence of 6.3 ± 0.5% glucose, 10.8 ± 0.2% fructose, and 38.2 ± 0.3% sucrose. When compared to established standard ranges, the sugar content of molasses aligns with the anticipated baseline parameters. The sucrose content ranges from 30 to 40%, showcasing the dominance of disaccharides in molasses. The measured values for fructose (5–12%) and glucose (4–9%) fall within the expected range, as reported by Teclu et al. (2009).

### 3.2 Microbial diversity of kombucha

**Figure 2:**
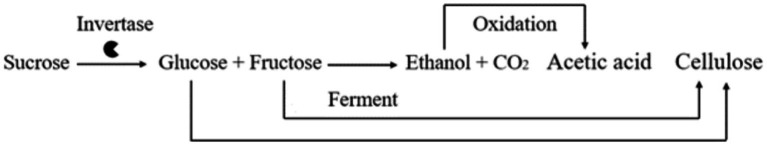
Kombucha metabolism during fermentation (Selvaraj and Gurumurthy, 2023).

**Figure 3:**
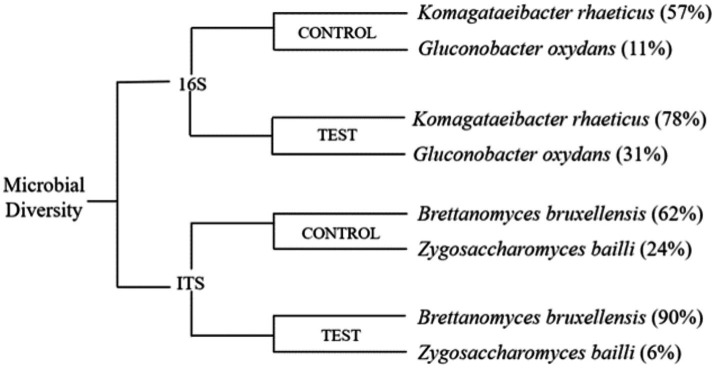
Dendrogram of the microbial diversity of kombucha tea.

Kombucha tea is generally comprised of osmophilic strains of yeast, such as *Brettanomyces* spp., *Candida* spp., *Lachancea* spp., *Pichia* spp., *Saccharomyces* spp., *Schizosaccharomyces* spp., *Zygosaccharomyces* spp., and acetic acid bacteria, including *Acetobacter* spp., *Gluconobacter* spp., *Gluconacetobacter* spp., *Komagataeibacter* spp., and *Lactobacillus* spp. (Teoh et al., 2004; Jayabalan et al., 2014). During kombucha fermentation, the yeast species in the culture produce invertase, which hydrolyzes sucrose into glucose and fructose and metabolizes ethanol. The obligate anaerobic acetic acid bacteria in the culture oxidize ethanol and excrete acetic acid (Figure 2) (Selvaraj and Gurumurthy, 2023). The samples were sequenced, and the quality was analysed using FastQC. The control sample from the black tea and sugar had (Figure 3) *Komagataeibacter rhaeticus* (57%) and *Gluconobacter oxydans* (11%) as the major bacterial species. The test sample from the tea dust and molasses had *Komagataeibacter rhaeticus* (78%) and *Gluconobacter oxydans* (31%) as the major bacterial species. The main fungal species in the control sample were *Brettanomyces bruxellensis* (62%), and *Zygosaccharomyces bailli* (24%). The main fungal species in the test sample were *Brettanomyces bruxellensis* (90%), and *Zygosaccharomyces bailli* (6%). The most commonly found bacterial species in the control and test samples are *Komagataeibacter rhaeticus* (Semjonovs et al., 2017; De Roos and De Vuyst, 2018; Machado et al., 2018; Gaggìa et al., 2019) and *Gluconobacter oxydans* (Greenwalt et al., 2000; Watawana et al., 2015; Chakravorty et al., 2016) and the yeast species in the control and the test samples are *Brettanomyces bruxellensis* (Liu et al., 1996; Teoh et al., 2004; Nguyen et al., 2015; Villarreal-Soto et al., 2018; Gaggìa et al., 2019) and *Zygosaccharomyces bailli* (Liu et al., 1996; Greenwalt et al., 2000; Teoh et al., 2004; Jayabalan et al., 2008a; Watawana et al., 2015; Villarreal-Soto et al., 2018; Gaggìa et al., 2019). Machado had studied that *Komagataeibacter rhaeticus* was able to produce bacterial cellulose using sugarcane-substituted culture medium as a carbon source (Machado et al., 2018). *K. rhaeticus* metabolizes the fructose and glucose to form cellulose (Rangaswamy et al., 2015). It oxidizes the ethanol to acetic acid by the alcohol dehydrogenase and acetaldehyde dehydrogenase enzymes (Nascimento et al., 2021). *Gluconobacter oxydans* is an acetic acid bacterium that is widely used in industries to produce acids like ascorbic acid, gluconic acid, aldehydes, and ketones like dihydroxyacetone from sugars and alcohols (Da Silva et al., 2022). *Brettanomyces bruxellensis* produces lactic acid, acetic acid, phenols and N-heterocyclic compounds; and tetrahydropyridines form lysine, which causes off flavors. *B. bruxellensis* is well known as a dietary supplement as it boosts the immune system through the probiotic effects that aid digestion and inflammation (Berbegal et al., 2018). *Zygosaccharomyces bailli* is fructophilic, fermenting fructose more favorably than glucose to produce alcohols, polyphenols, and fatty acids. *Z. bailli* produces CO_2_ and acetic acid and is resistant to ethanol (Fernandes et al., 1997). The *Komagataeibacter* strains are well-known for their ability to efficiently metabolize ethanol through a process known as ethanol oxidation. This metabolic pathway allows them to convert ethanol into acetic acid (Nascimento et al., 2021). The ethanol present in both the control and test samples undergoes metabolism, resulting in the production of acetic acid.

### 3.3 Biochemical analysis and antioxidant activity of kombucha

#### 3.3.1 Estimation of acid content in kombucha tea

**Figure 4:**
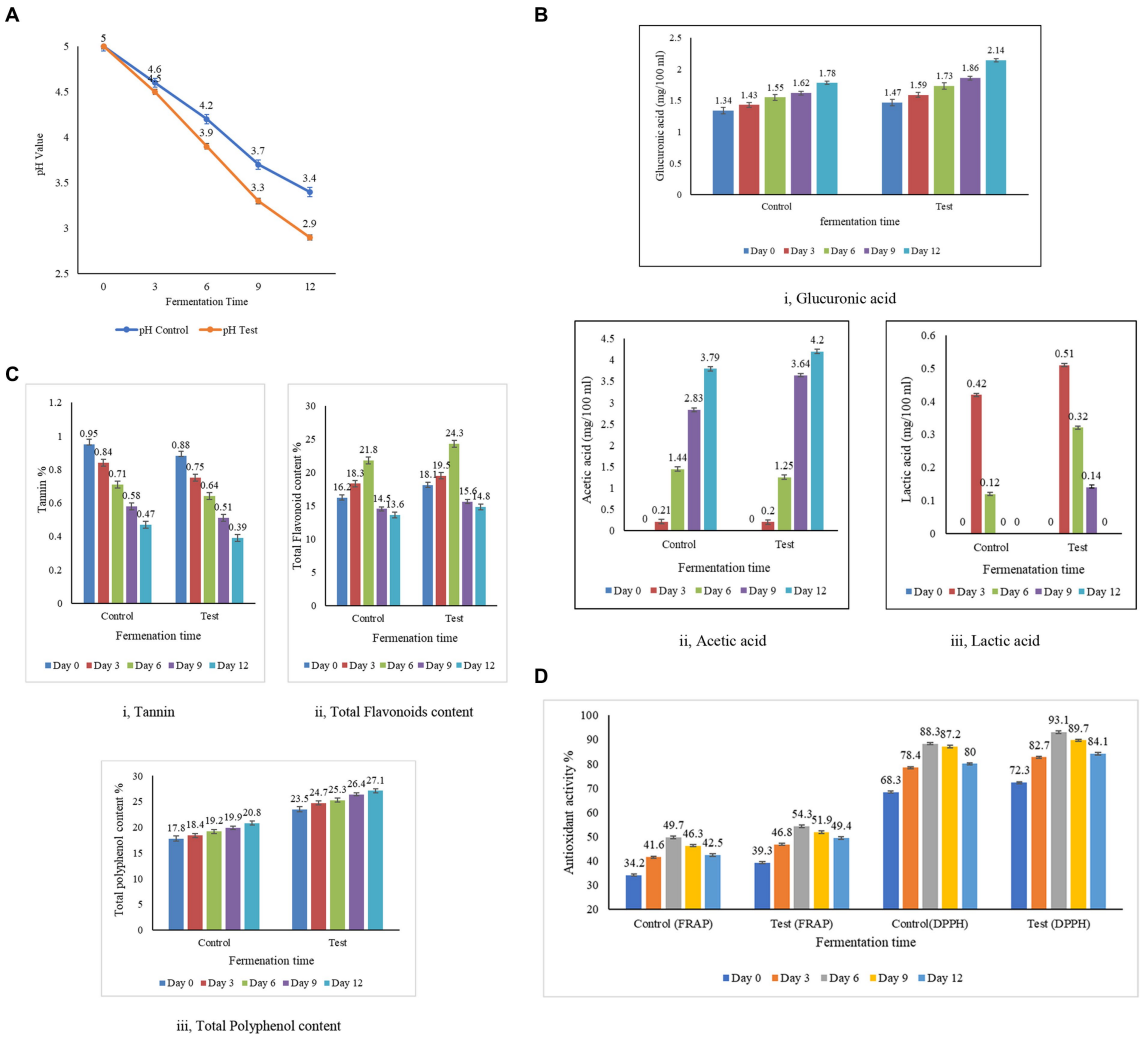
Biochemical analysis and antioxidant activity of kombucha tea from the test and the control samples. **(A)** Estimation of pH in the test and the control samples. **(B)** Estimation of acid content in the test and the control samples. **(C)** Estimation of tannin, flavonoid, and total polyphenol content of the test and the control samples. **(D)** Antioxidant activity by the DPPH method and the FRAP method in the test and the control samples.

Acidity is a natural preservative that inhibits the growth of harmful microorganisms and is also responsible for the sour taste of kombucha tea. The concentrations of acetic and glucuronic acids in the test samples were consistently high in the control sample throughout the entire 12 day fermentation period (Figure 4B). The acid content in the samples demonstrates a proportional relationship with the duration of fermentation, with an extension in fermentation days leading to an increase in acid levels (Jayabalan et al., 2007). The citric acid content was not detectable on any day between 1 and 18 days of fermentation (Jayabalan et al., 2007). Similarly, the levels of gluconic acid and citric acid in the control and the test were below the detection level, which is less than 0.1 mg/100 mL. The lactic acid content was not detectable on any day between 1 and 10 days of fermentation (Neffe-Skocińska et al., 2017). Similarly, the lactic acid levels were in a detectable range after day 9 in the control sample and day 12 in the test sample. However, a trace amount of lactic acid was identified in the test sample, likely attributed to the presence of molasses. Malbaša et al. (2008) have studied that the lactic acid content in molasses is naturally higher. In the test sample, the acetic acid concentration is measured at 4.20 ± 0.02%, while in the control sample, it is recorded at 3.79 ± 0.02%. The lactic acid content in the test sample is higher than the control sample, as the dominance of *Saccharomyces* is high in the test sample. The enhanced production of acetic acid in the test sample is linked to the greater prevalence of the *Gluconobacter* and *Komagataeibacter* strains, which account for 31 and 78%, respectively. This is in contrast to the control sample, where the dominance of these strains is lower, at 11 and 57%, respectively. The acetic and glucuronic acid concentrations in both the control and test samples initially started at low levels, but as fermentation progressed, higher concentrations of acids were observed. This increase can be attributed to the hydrolysis of sugar by yeast in the culture, followed by the conversion of sugars into acids by bacteria, as highlighted by the study conducted by Selvaraj and Gurumurthy (2023). The meticulous analysis of acid profiles in kombucha fermentation reveals dynamic changes in acetic, glucuronic, citric, and lactic acids, each contributing uniquely to the overall composition and flavor profile of the fermented beverage. The sugar substrates are utilized, creating an increase in the production of pH and organic acids throughout the fermentation period, along with a reduction in alcohol. The interplay of these acids is a testament to the complex biochemical processes that occur during kombucha fermentation.

#### 3.3.2 Estimation of tannin, flavonoid, and total polyphenol content in kombucha tea

During fermentation, the tannins decrease while the phenols increase, indicating the dynamic nature of the biochemical transformations that occur during prolonged fermentation (Hawashi et al., 2019). In contrast, the flavonoid content increases as the polyphenols increase, contributing to the augmentation of kombucha’s reducing power as the fermentation duration extends. The colour changed from dark brown to light brown with the progress of fermentation (Chakravorty et al., 2016). The transition from yeast to lactic acid bacteria dominance on the 6th day of fermentation indicates that the increased diversity of microorganisms significantly contributes to the enhanced antioxidant properties of kombucha tea (Chakravorty et al., 2016). Subsequently, after the sixth day of fermentation, the growing dominance of lactic acid bacteria leads to the release of enzymes that break down polyphenols, resulting in a decline in flavonoids—a subgroup of polyphenols. This process contributes to the reduction of antioxidant activity in the kombucha tea after day 7 of fermentation (Christiani Dwiputri and Lauda Feroniasanti, 2019). Tannins, polyphenolic compounds found abundantly in tea leaves, play a significant role in the flavor and properties of tea. The analysis of tannin content in both the control and test samples of kombucha tea, as presented in Figure 4C-i, reveals intriguing insights into the composition of these samples. The recorded tannin content in the control sample demonstrates a range from 0.47 to 0.95%, while the test sample exhibits a range varying from 0.39 to 0.88%. Notably, the tannin content in the control sample is higher than that in the test sample. This suggests that the tannin content of the tea leaves used in the control is higher than that of the tea dust employed in the test sample. The microbial activity within the SCOBY (Symbiotic Culture of Bacteria and Yeast) layer, a crucial component of the kombucha fermentation process, is highlighted as a transformative force. The breakdown of tannins by these microbes results in the generation of catechins and gallic acid, as detailed in the study by Muzaifa (Muzaifa et al., 2023). Catechins and gallic acid are bioactive compounds known for their potential health benefits and are considered valuable contributors to the overall antioxidant capacity of tea (Ivanišová et al., 2020). The observed differences in tannin content between the control and test samples emphasize the dynamic nature of the fermentation process and the role of microbial activity in modifying the chemical composition of the tea. This information sheds light on the intricate interplay between tannins, microbial transformation, and the resultant generation of beneficial compounds, enriching our understanding of the complex biochemical processes underlying kombucha fermentation. As the fermentation process of kombucha tea progresses, a noticeable shift in its pH indicates changes in the composition of flavonoids within the sample, as documented by Jakubczyk et al. (2020). Flavonoids, being phenolic compounds, play a crucial role in conferring antioxidant properties to tea. The flavonoids in the control and test samples increased from day 0 to day 6, as indicated in Figure 4C-ii, wherein the microbial activity enhanced the concentration of flavonoids, reinforcing the antioxidant potential of the tea. After the 6th day of fermentation, there is an inclination in the content of flavonoids in both samples. This inclination suggests that the fermentation process, as it matures, continues to foster the accumulation of flavonoids, emphasizing the intricate relationship between fermentation duration and the composition of bioactive compounds (Jakubczyk et al., 2020). The observed shift in pH and the parallel increase in flavonoid content during the fermentation of kombucha underscore the dynamic interplay between microbial activity and the enhancement of antioxidant properties. These findings contribute to a deeper understanding of the temporal changes in the composition of flavonoids, during kombucha fermentation. The correlation between color intensity and phenolic content becomes evident, with the high intensity of blue color serving as an indicator of elevated phenolic value, a metric directly associated with antioxidant activity. According to the findings presented in Figure 4C-iii, both the control and test samples exhibit an increase in phenolic content with prolonged fermentation periods. This aligns with the observations made by Chakravorty et al. (2016), highlighting a positive correlation between polyphenol levels and the duration of fermentation. The microorganisms break down complex polyphenols into smaller molecules in the course of fermentation, potentially leading to an elevation in the overall phenolic content in the kombucha sample, as investigated by Ivanišová et al. (2020). Blackstrap molasses emerges as a significant contributor to the polyphenolic richness of the samples. The control samples, utilizing molasses, display a higher total phenolic content compared to the test samples. This disparity is attributed to the fact that black strap molasses, employed in the test samples, is a superior source of polyphenols when compared to sugarcane juice and syrup, as emphasized by Guan et al. (2014). Kombucha, renowned for its high polyphenol content and robust antioxidant activity, further substantiates these findings. Both Alejandra and Villarreal (Villarreal-Soto et al., 2019; Alejandra Villarreal-Soto et al., 2020) have attested to the elevated polyphenolic and antioxidant properties associated with kombucha, affirming its status as a valuable functional beverage with potential health benefits.

#### 3.3.3 Antioxidant activity by the DPPH method and the FRAP method in kombucha tea

The fermentation process, catalyzed by microbial biotransformation, has been recognized as a key contributor to enhancing antioxidant activity (Hu et al., 2020). DPPH and FRAP methods were used to assess antioxidant activity, enhancing the reliability and robustness of the analysis. DPPH evaluated the sample’s ability to neutralize free radicals, while FRAP measured its reducing capacity. This comprehensive approach ensured a more thorough understanding of the sample’s antioxidant potential, validated the results, and provided a more robust assessment of its antioxidant activity. The results of the analysis of the antioxidant potential of the control sample (black tea and sugar) revealed a range between 68.3 ± 0.2% and 88.3 ± 0.1% DPPH inhibition. In contrast, the antioxidant potential of the test sample (tea dust and molasses) revealed a range between 72.3 ± 0.2% and 93.1 ± 0.2% DPPH inhibition. The substituted substrate (test) displayed a progressive increase in antioxidant potential from days 1 to 6, reaching its peak on day 6 at 93.1 ± 0.2%. However, beyond day 6, the antioxidant activity showed a decreasing trend, emphasizing the importance of monitoring fermentation duration. This observed peak in antioxidant activity on day 6 aligns with findings by Jakubczyk et al. (2020), who reported the highest antioxidant activity in kombucha from day 1 to day 7. The green tea displayed an antioxidant activity of 94.61% ± 1.29 on the first day and 91.40 ± 0.57 on day 7 (Jakubczyk et al., 2020), and this level of activity closely resembled the antioxidant activity observed in our experimental kombucha made from tea dust and molasses on the sixth day. The antioxidant activity was measured using the ferric ion reducing antioxidant power (FRAP) assay for the control samples with black tea and sugar, and the test samples with tea dust and molasses. The analysis of the antioxidant potential in the control sample (black tea and sugar) indicated a range between 34.2 ± 0.3% and 49.7 ± 0.3%. Conversely, the test sample (tea dust and molasses) demonstrated an antioxidant potential ranging from 39.3 ± 0.1% to 54.1 ± 0.4%. The Free Radical-Scavenging Activity (FRAP) values for both the control and test samples of kombucha tea exhibited a rise during the fermentation period, followed by a distinct decrease after the 6th day. This trend aligns with the findings documented in the study conducted by Gaggia and Jakubczyk (Gaggìa et al., 2019; Jakubczyk et al., 2020). The kombucha’s antioxidant activity, assessed by FRAP and DPPH methods, consistently showed that test samples prepared with tea dust and molasses outperformed control samples over the 12 day duration. This indicated enhanced antioxidant activity compared to black tea and sugar. Both methods revealed a consistent trend of higher antioxidant activity in test samples, with a similar magnitude of difference compared to control samples. The test samples consistently exhibit higher antioxidant activity compared to the control samples across the 5 days. This indicates that the combination of tea dust and molasses contributes to enhanced antioxidant potential compared to the black tea and sugar combination in the control samples. The correlation analysis revealed statistically significant relationships between tannin, flavonoid, phenol levels and antioxidant activity (Figure 4). The varied correlation patterns between tea leaves and tea dust over the fermentation period highlight the dynamic impact of raw materials on kombucha composition. Kombucha tea derived from agro-industrial waste, as demonstrated by the test sample, exhibited significantly higher activity compared to traditionally prepared tea with a high statistical significance (***p > 0.001 at 99.9%). The data are expressed as the mean ± SD, where the number of samples was 3 (*n* = 3 samples). The change in raw materials, particularly the incorporation of tea dust and molasses, significantly influenced the antioxidant activity of the test sample. The study aligns with previous research, indicating an inverse relationship between antioxidant activity and fermentation time (Jakubczyk et al., 2020). Comparing the test (tea dust and molasses) with the control (black tea and sugar) on all 12 days revealed the test sample’s potentially higher antioxidant activity. The correlations with vitamin C production and acids further emphasize the multifaceted nature of factors influencing antioxidant activity in kombucha (Malbaša et al., 2011). These findings underscore the impact of raw material selection and fermentation duration on the antioxidant properties of kombucha, highlighting the potential of kombucha made from tea dust and molasses as a rich source of antioxidants.

### 3.4 Nutritional profiling of kombucha

The nutritional assessment of kombucha fermentation was conducted on day 6, a time point when antioxidant levels were observed to be higher in both the control and test samples. The nutritional composition of kombucha tea, produced from black tea and sugar (control) and tea dust and molasses (test), was thoroughly analyzed (refer to Table 2). The content of saturated fat, trans fat, protein, cholesterol, dietary fiber, ethanol, copper, and vitamin A in the control and test samples was determined to be below the detection level (BDL), measuring less than 0.1 g/100 mL and undetectable using the analytical methods applied at the specific time point of measurement. The increase in acetic acid production during fermentation leads to the demolishment of the alcohol in the kombucha samples during fermentation (Jayabalan et al., 2010). Notably, the content of carbohydrates, sodium, calcium, iron, and potassium was found to be higher in the test sample compared to the control, although still within acceptable limits. While the test sample exhibited elevated levels of these nutritional components, they remained below the critical limit, indicating that the nutritional profile of the test sample falls within the range deemed suitable for human consumption. This underscores the positive nutritional attributes of kombucha produced with tea dust and molasses, affirming its potential as a nutritious beverage option. Blackstrap molasses stands out for its richness in sugars, encompassing sucrose, glucose, and fructose. The glucose and fructose levels of the test samples are higher than the control samples and are statistically significant. Specifically, the glucose level in the control is recorded at 0.6 ± 0.1 g/100 mL, while in the test, it reaches 0.7 ± 0.1 g/100 mL. Similarly, the fructose content in the control is noted as 0.1 ± 0.05 g/100 mL, whereas in the test, it elevates to 0.4 ± 0.1 g/100 mL. Notably, the sucrose level in the test sample is substantially higher at 1.4 ± 0.1 g/100 mL compared to the control sample, which registers at 0.7 ± 0.1 g/100 mL, and this difference is highly significant. The elevated sugar content in the test sample can be attributed to a slower fermentation rate than in the control sample. During the fermentation process, yeast in the culture hydrolyzes sucrose into glucose and fructose, leading to a gradual decrease in sugar levels as the fermentation days progress. This intricate interplay between fermentation dynamics and sugar composition underscores the complexity of the biochemical transformations occurring in kombucha, particularly when utilizing substrates like blackstrap molasses. Molasses, being inherently rich in minerals, contributed significantly to the high mineral content observed in the test sample compared to the control sample. The metabolic activity of kombucha and the SCOBY (Symbiotic Culture of Bacteria and Yeast) plays a pivotal role in metabolizing minerals, as emphasized by Bauer-Petrovska and Petrushevska-Tozi (2000). The mineral composition of calcium, iron, and potassium in the test sample was notably higher at 19.4 ± 0.15, 23.1 ± 0.25, and 28.3 ± 0.25 mg/100 mL, respectively, in comparison to the control sample with values of 1.6 ± 0.3, 1.1 ± 0.2, and 2.4 ± 0.2 mg/100 mL, respectively. It is essential to highlight that the copper levels in both the control and test samples were below the detection level (BDL), measuring less than 0.1 g/100 mL. The levels of iron, potassium, and calcium in the test sample, compared to the control, are highly statistically significant and can be attributed to the presence of molasses in the test sample. The influence of molasses contributes significantly to the mineral enrichment of the test sample, showcasing the impact of varied substrates on the mineral composition of kombucha. Molasses, as established by Jamir et al. (2021), is known for its abundance of essential vitamins such as B2, B6, and B12. In parallel, kombucha tea, as noted by Bauer-Petrovska and Petrushevska-Tozi (2000), is recognized for its richness in vitamins B and C. In the analysis of both the control and test samples, it was observed that the vitamin A levels were below the detection level (BDL), measuring less than 0.1 g/100 mL. Examining the control sample revealed that the levels of vitamins B2 and B3 were below the detection level (BDL), specifically less than 0.1 mg/100 mL. In contrast, the test sample exhibited detectable levels of vitamin B2 at 0.30 ± 0.02 mg/100 mL and vitamin B3 at 0.33 ± 0.02 mg/100 mL. The inclusion of molasses in the test sample significantly contributed to the improvement of the overall vitamin content, rendering it more nutritionally enriched. The test sample displayed higher levels of vitamins B1, B6, B9, B12, and C compared to the control sample, and this difference was found to be statistically significant (*p* > 0.1). Research conducted by Malbaša et al. (2011) highlighted that the levels of vitamin B2 (9.60 mg/100 mL) and vitamin C (28.98 mg/L) in green tea are higher on day 10 of fermentation than in native kombucha made from black tea. This underscores the dynamic nature of the biochemical properties of kombucha, which are influenced by nitrogenous compounds derived from tea waste. These compounds contribute to the vitamin composition, providing valuable insights into the nutritional attributes of kombucha.

**Table 2:** Nutritional labelling of kombucha tea from control (black tea and sugar) and test (tea dust and molasses).

S. No	Parameters	Unit	Sample A (Control)	Sample B (Test)
1	Saturated fat	g/100 mL	BDL (DL:1.0)	BDL (DL:1.0)
2	Trans fat	g/100 mL	BDL (DL:1.0)	BDL (DL:1.0)
3	Protein	g/100 mL	BDL (DL:1.0)	BDL (DL:1.0)
4	Cholesterol	g/100 mL	BDL (DL:1.0)	BDL (DL:1.0)
5	Dietary fiber	g/100 mL	BDL (DL:1.0)	BDL (DL:1.0)
6	Ethanol	g/100 mL	BDL (DL:1.0)	BDL (DL:1.0)
7	Sodium	mg/100 mL	2.95 ± 0.15	4.35 ± 0.25
8	Calories	Kcal/100 mL	6.64 ± 0.32	14.85 ± 0.25
9	Carbohydrates	g/100 mL	1.41 ± 0.18	3.135 ± 0.12
Minerals				
1	Calcium	mg/100 mL	1.6 ± 0.30	19.4 ± 0.15
2	Iron	mg/100 mL	1.1 ± 0.20	23.1 ± 0.25
3	Potassium	mg/100 mL	2.4 ± 0.20	28.3 ± 0.25
4	Copper	mg/100 mL	BDL (DL:1.0)	BDL (DL:1.0)
Sugars				
1	Fructose	g/100 mL	0.1 ± 0.05	0.4 ± 0.1
2	Glucose	g/100 mL	0.6 ± 0.1	0.7 ± 0.1
3	Sucrose	g/100 mL	0.7 ± 0.1	1.4 ± 0.1
Vitamins				
1	Vitamin A	mg/100 mL	BDL (DL:0.1)	BDL (DL:1.0)
2	Vitamin B1	mg/100 mL	0.42 ± 0.01	0.58 ± 0.01
3	Vitamin B2	mg/100 mL	BDL (DL:0.1)	0.30 ± 0.02
4	Vitamin B3	mg/100 mL	BDL (DL:0.1)	0.33 ± 0.02
5	Vitamin B6	mg/100 mL	0.44 ± 0.01	0.75 ± 0.02
6	Vitamin B9	mg/100 mL	0.12 ± 0.01	0.19 ± 0.03
7	Vitamin B12	mg/100 mL	0.8 ± 0.01	0.9 ± 0.03
8	Vitamin C	mg/100 mL	1.26 ± 0.02	1.38 ± 0.06

BDL, below the detection level; DL, detection level which is less than 0.1 mg/100 mL.

### 3.5 Sensory analysis

#### 3.5.1 Coliform test

A coliform test was done with the membrane filter method to check for the presence of any pathogenic fecal coliforms. The test was negative; the absence of organisms indicates that it is safe for human consumption (Abu-Sini et al., 2023). Hence, the samples were further tested for organoleptic properties by sensory evaluation.

#### 3.5.2 Evaluation of organoleptic properties

**Figure 5:**
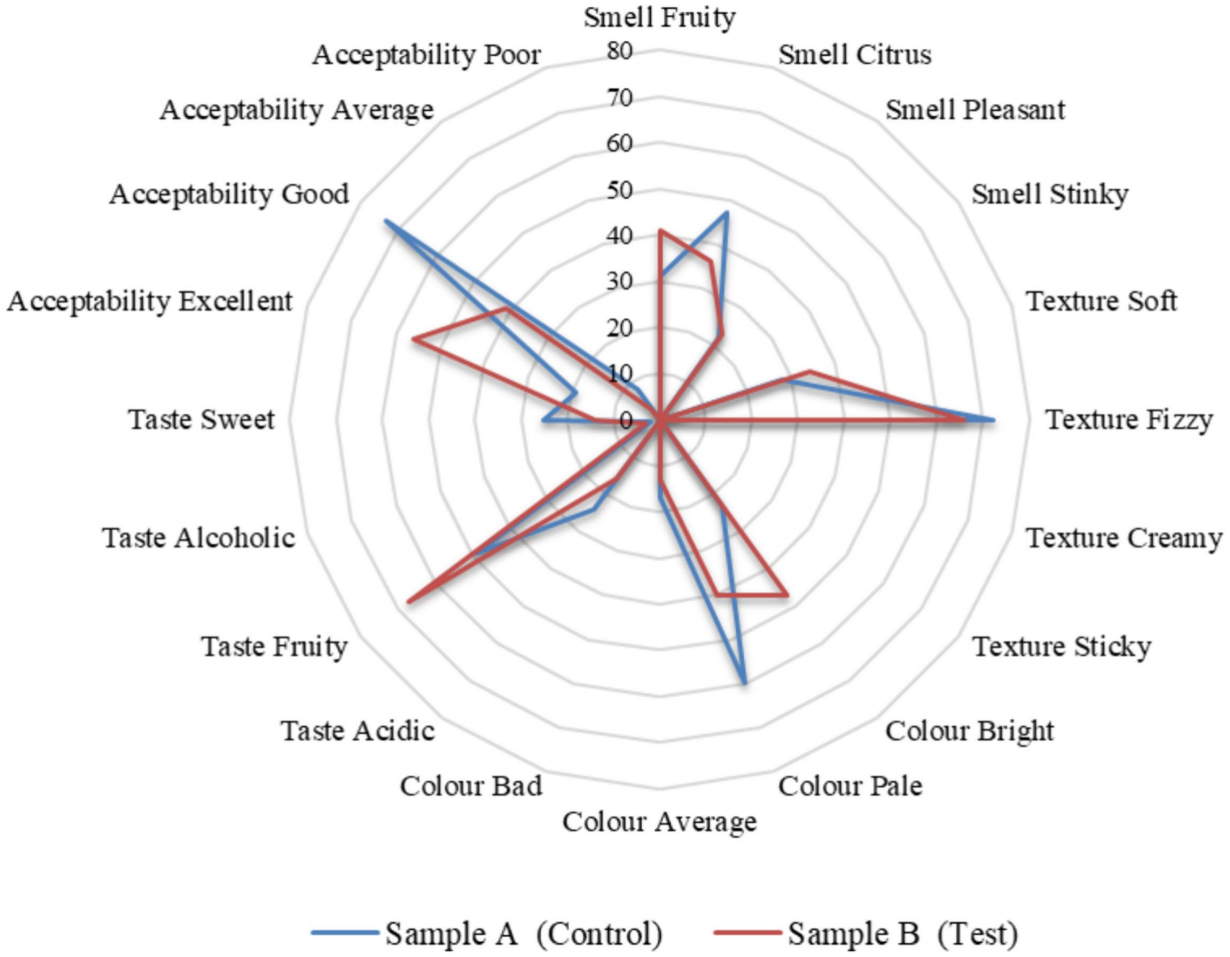
Sensory analyses of kombucha tea from the control (black tea and sugar) and test (tea dust and molasses).

The organoleptic properties are examined through the senses- taste, sight, smell, and touch of individual experiences with tea samples (Figure 5). Sensory analysis is done with the texture (soft, fizzy, creamy, and sticky), the smell (fruity, citrus, pleasant, and stinky), the color (bright, pale, average, and bad), the taste (alcoholic, fruity, acidic, and bad), and the acceptability (excellent, good, average, and poor) of kombucha from the control (black tea and sugar) and the test (tea dust and molasses). Kombucha beverages obtained from the test sample were citrusy, fizzy, bright, fruity, and good. The kombucha made from black tea and sugar (the control) had a smell of 22% pleasant, 31% fruity, and 47% citrus as dominant, but was not stinky at all. The texture was 28% soft and 72% fizzy, but not creamy 0% or sticky 0%. Color: 23% bright, 60% pale, 17% average but not bad, 0%. Taste: 24% acidic, 49% fruity, 2% alcoholic, and 25% sweet. The overall acceptability was 19% excellent, 72% good, and 8% average, but not poor, 0%. The kombucha made from tea dust and molasses (tested) had a smell of 23% pleasant, 36% citrus, and 41% fruity, and was dominant but not stinky at all. The texture was 34% soft and 66% fizzy, but not creamy (0%) or sticky (0%). Color: 47% are bright, 40% are pale, and 13% are average but not bad 0%. Taste: 16% acidic, 67% fruity, 3% alcoholic, and 14% sweet. The overall acceptability was 56% excellent, 41% good, and 3% average, but not poor (0%). Kombucha beverages obtained from the control sample attracted consumers because of their fruity taste, fizz texture, bright color, and fruity smell. The overall acceptability was given as excellent. The results show that the control and test samples are almost similar in texture (fizzy), color (bright), and taste (fruity). The smell of the samples was different, as the test had a citrus odor and the control had a fruity odor. This is because the sweetness in the molasses provided a fruity odor. The overall acceptability of the test and the control was also good for the test and excellent for the control. The preference of the consumer was also noted: out of 100 people, 12 thought A was better than B, and 88 thought B was better than A. The test sample (substituted substrates) had good sensory properties and an approval score compared to the control sample. The kombucha made from yarrow extract was acidic and had a pleasant taste and odour (Vitas et al., 2018). Neffe-Skocińska et al. (2017) showed the average quality of the kombucha and that the smell of tea, and sour taste were detected with high intensity. Ivanišová et al. (2020) have prepared fresh kombucha with a pleasant smell and a sour to fruity taste. The kombucha tastes sour due to the reduction in pH. The kombucha from the control and test samples was rated good and excellent, respectively. A high score for smell and taste correlates with the fruity, acidic, and citrus flavours of the sample. The texture and colour were mostly fizzy and pale, respectively. The samples are fizzy in texture, non-alcoholic (<0.5%), and carbonated. This is because the yeast in the kombucha produces alcohol and carbon dioxide. The tannins in the kombucha make it darker and paler the longer you brew it. The acetic acid bacteria *Komagataeibacter rhaeticus* and *Gluconobacter oxydans* produced acetic acid, which gave kombucha its tangy flavor and fruity aroma (Bishop et al., 2022). They also produced CO2, which was responsible for the fizzy profile of the kombucha drink. The yeast strains *Brettanomyces bruxellensis* and *Zygosaccharomyces bailii* produced fruity esters and phenols that enhanced the fruity smell and flavor (Bishop et al., 2022). The approval of the test sample is determined by the overall preference of the consumers. The sensory properties of kombucha from both raw materials were comparatively good and acceptable.

## 4 Conclusion

Traditionally, kombucha is prepared with black tea and table sugar, chosen as the control sample. In contrast, the substituted raw materials, tea dust, and molasses are taken as test samples and compared for further experimental analysis. The microbial diversity of kombucha, characterized by the presence of yeast and acetic acid bacteria, was explored through sequencing and analysis. The predominant microbial species identified in both control and test samples included *Komagataeibacter rhaeticus, Gluconobacter oxydans, Brettanomyces bruxellensis*, and *Zygosaccharomyces bailli*, with varying dominance levels for each organism as they followed a common fermentation pathway. These microorganisms play essential roles in metabolizing sugars, producing acids, and contributing to the unique flavor profile of kombucha. Biochemical analysis revealed dynamic changes in acid content during the fermentation period, with a proportional increase in acetic and glucuronic acids. The test sample exhibited higher levels of acetic acid, attributed to the dominance of *Gluconobacter* and *Komagataeibacter* strains. Tannin, flavonoid, and total polyphenol content showed variations during fermentation, with the test sample consistently displaying higher antioxidant potential compared to the control. The outcomes of kombucha’s antioxidant activity were consistent across both FRAP and DPPH assays, showing an increase from day 1 to 6 and subsequent declines. Consumers, being health-conscious in terms of diet and intake, have recently shown increased interest in probiotic drinks. A proper sensory evaluation study was conducted to provide a clear view of flavor profiles and consumer acceptance. The test sample attracted consumers with its fruity smell, fizzy texture, bright color, and fruity taste, resulting in a noticeably excellent acceptability rate. The nutritional properties of kombucha are an additional benefit to the consumer beyond their overall liking and preference. The microbes produced metabolites like acetic acid, phenols, and CO_2_, resulting in a tangy and carbonated taste with a fruity aroma. A unique organoleptic profile was achieved by the microbial synergy of the microorganisms and their metabolic activity. Nutritional profiling indicated that kombucha produced from tea dust and molasses had elevated levels of minerals, carbohydrates, and vitamins compared to the control. The inclusion of molasses contributed to increased sugar content and enhanced mineral composition, showcasing the nutritional richness of the test sample. The antioxidant activity, organoleptic properties, and nutritional profiling of kombucha fermentation from molasses are remarkably high and statistically significant in the kombucha tea sample with tea dust and molasses. The research presented here achieved mass production of kombucha probiotic drink from cost-effective substitutes. In a comparative analysis, it becomes apparent that the test sample excels beyond the control sample, particularly in terms of bioactive and nutritional compounds. The impact of raw material selection, fermentation duration, and microbial activity on the microbial, biochemical, nutritional, and sensory aspects of kombucha have been studied in detail. The utilization of tea dust and molasses as alternative substrates demonstrated potential benefits in terms of microbial diversity, antioxidant activity, and nutritional enrichment, presenting opportunities for the development of novel kombucha formulations. This work has propelled and levelled up the insight for the production of economically feasible and nutrition-rich kombucha tea on a large scale. The research outcomes not only contribute to a deeper understanding of the interplay between relationships among raw materials, microbial composition, and bioactive compounds but also shed light on the resulting nutritional value, ensuring acceptable sensory attributes. The emerging kombucha brewing industry can leverage our study’s evidence-based insights on raw material selection, fermentation dynamics, and product quality optimization to develop novel formulations with improved nutrition, antioxidants, and consumer appeal. The challenges and future perspectives encompass key considerations such as standardizing and scaling up kombucha production, exploring novel ingredients as raw materials with health and safety considerations, consumer education, market acceptance, and the environmental sustainability of kombucha products.

## Data availability statement

The datasets presented in this study can be found in online repositories. The names of the repository/repositories and accession number(s) can be found in the article/supplementary material.

## Author contributions

SS: Writing – original draft, Software, Methodology, Investigation, Formal analysis, Data curation. KG: Writing – review & editing, Visualization, Validation, Supervision, Resources, Project administration, Conceptualization.
